# Multi-Level Governance of COVID-19 Pandemic and the Solitude Within Geriatric Oncology

**DOI:** 10.3389/fpubh.2021.647444

**Published:** 2021-04-29

**Authors:** Vasco Lourenço Fonseca, Joaquim Croca Caeiro, Rui Miranda Juliao

**Affiliations:** ^1^Centre of Public Administration and Public Policies, Institute of Social and Political Sciences, Universidade de Lisboa, Lisboa, Portugal; ^2^Centro Hospitalar de Lisboa Ocidental, E.P.E., Lisboa, Portugal

**Keywords:** governance, solitude, geriatric oncology, public health, cancer, COVID−19

Present worldwide governance deals with people's effectively and justly performance (1). Nowadays, governance presumes a social constructionist strategy led by the government. As the central policymaker, a nation's state administration decides social and economic resources towards its development. This faculty of formulating, planning, and fulfilling tasks coordinate the nature of rule patterns. It gives place to new governing practices that require accurate initiatives, from which emerge new theories that give birth to different dilemmas. The relationship between state and societies changes once the governing activity works with non-governmental organizations, like private companies and non-profitable service providers (2).

Global governance is a set of regulations developed to answer specific issues, national or regional, or to supply transnational common goods (3). It also refers to multilevel governance when tasks are separated by sector and not by level, which results in a highly concentrated network of outstanding quality international and transnational institutions. These are far more intrusive than conventional international ones. On the one hand, they can resist national political governance by the decision-making of the majority. On the other, they can sort out disputes both by transnational means' monitoring and knowledge control and interpretation (3). In the globalization era, nations favour global organisations since are commonly accepted and have the authority to decide for millions of people. Simultaneously, they restrict international regulation based on the national sovereignty and their vetting ability.

Public and private organisations have their own administration. As a rule, management includes not only decision-making but also social intervention. We usually include the political element, which is extremely relevant for public administration. This is the most substantial difference from private organisations. The Public Administration Sciences acknowledges plus providers of goods and services towards the satisfaction of the communities (3). Additionally, they check the public policies arrangement to resolve difficulties, by using the resources available. Although significant differences, public administration answers to political government. While the public administration, despite some unreasonable behaviours and theories, relies on a regulated, objective and scientific thought, governmental politics is more subjective and intuitive, and less formal (3).

Circulating everywhere, most people contaminated by the newly discovered coronavirus (COVID-19) will experience mild to moderate respiratory illness and recover well. Others, especially the cardiovascular, diabetes, chronic respiratory and cancer ones, are more likely to develop severe illness (4). Elderly and cancer patients are at increased risk of obtaining COVID-19 and dying from it. Moreover, older people with cancer are at the highest risk of being excluded from intensive care support for COVID-19 infection and adequate cancer treatment if resources are restricted. Global improvement in health care and living conditions has generated an ageing population, and, by 2020, we had more than 700 million at an advancing age and high-risk cancer factor worldwide (5). Likewise, loneliness is a known risk factor for poor mental and physical health outcomes and quality of life in the general population, and preliminary research suggests that loneliness relates to poorer health outcomes in cancer patients (6).

Leadership is essential for governance; it bonds reputation to performance. Isabel Fonseca defends that despite society's perception of ethical politic behaviour, the media can either amplify or lessen it (7).

Ancient philosophy's ethics embraced both social behaviour and a high living standard and, politically and jurisdictionally, ought to be sovereign to survive (8). Economically speaking in politics, the most important for a liberal democracy was to assure society's endurance. As per Adam Smith's “invisible hand,” the stock market is essential to aid the worlds progression with minimal national intervention. In other words, if the stock market shuts down, our economic model collapses (9). Medical ethical principles were inspired on a scientific model created during the era of Illuminism, and later developed into the present biomedical model. For therapeutic specialists, bioethics means to make the best evidence-based practice available to every patient. Presently, the commonly called oriented treatment is considered the most accurate. However, it doesn't consider the social condition of patients (10), although we presume everybody has equal access to the most advanced treatment. Yet, the health model used in the East is based on combating diseases and promoting health. Lalonde, a prominent supporter, advocated that better health outcomes is achieved through several healthcare programs like vaccination prevention and sanitary improvements, which in turn has a holistic approach (11, 12). Vaccination is a biosocial approach for a sustainable health procedure. It is also a political determinant because it results from a political decision and strategy. Group immunization is accomplished by mass vaccination and is the best health policy to handle pandemics. Vaccines have proved to be safe, and the elderly with cancer shall be a priority. As a result of a politic policy executed by public administration, this biosocial strategy will contribute to social and health progress (13).

The current health systems are based on the biomedical model and scientific evidence with an intricate hospital practice approach (12). The World Health Organization (WHO), national governments and medical scientific communities have a unique role in the governance of health systems, in scientific knowledge communication and implementation of good health practices (4).

WHO plays a vital role in global health systems governance by establishing values seemingly familiar to every country, helping to regulate health policies. Some relate to a western perspective on the world, like individual freedom and science, which are considered key factors in a nation's development. Such multilevel governance might need to reflect on incorporating other cultures' elements to ensure a common understanding, like China's holistic approach to health care (12). The national health systems will gradually integrate these, while WHO will face the challenge of a global world that needs to both respect cultural distinctions and focus on its development.

In 2020, due to COVID-19, health systems face enormous challenges and demands. It became clear that we cannot approach this pandemic using the current complex approach model. COVID-19 awakened the global population towards local health organisations in general (authorities, hospitals, and local primary healthcare providers). It forced health systems worldwide to adapt their assistance to the population. In some instances, national decision-makers have chosen public policies according to the prevailing classic paradigm (14).

The WHO guidelines supersede national policies and limit public health policies. The pandemic health comebacks have been implemented through a multilevel government approach governance (15, 16). The strategy and efficiency of such answer varies between countries within the European Union territory, which proves that the political power still prevails over health specialists and professionals (1, 16).

As a response to the pandemic in Portugal, the WHO acting above the national level, and the DGS - Direção Geral de Saúde (National Health Administration) acting at a national level, are the driving forces for public health. Citizens know that scientists inform politicians with specific data, reports, scenario analyses and solutions in the current emergency state in healthcare. There are frequent meetings and the information is regularly made public (17).

The implementation of public health measures (personal protective equipment), isolation and social distance, are major solutions. Social isolation and the subsequent procedures for social distance like online working, is changing the relationship between healthcare professionals and their patients. Telemedicine increased, and so did solitude and its negative impact on physical and mental health. Despite being a growing issue in our society, the elderly has been the most affected (18). As social bonds decrease, the risk of loneliness goes up, and loneliness affect people from different socioeconomic backgrounds and age. Although regarded as a healthcare problem, solitude is still stigmatised, ostracised, and even ignored in some cultures (6).

Solitude is a risk factor for mental and neurologic diseases like depression and cognitive disorders (6). It is also related to the immune system, sleep disorders, pain, tiredness, and cancer (5). The bidirectional relationship between cancer and solitude subsists, mainly because cancer can contribute to loneliness. On the one hand, patients with cancer hold either more significant apprehensions about existence or unrealistic expectations. On the other, they fear to share their perceptions. Encouraged, seemingly, by relatives, some of the patients reveal constrained social behaviours like blame, shame or avoidance mainly when the subject is cancer, leading to seclusion behaviours. There are several social determinants like the fragility of the geriatric patient derived from isolation or frequently living in a senior residence, away from family and friends (5). The patient with cancer has an increased bias to loneliness due to age, debility, and his own experience of dealing with the disease. Administration boards for health institutions must deliver a specific social intervention to combat seclusion and negative expectations towards cancer (5, 6). The cancer patient belongs to the high-risk group of COVID-19 and needs specific answers according to the European Society of Medical Oncology (ESMO) and the International Society of Geriatric Oncology (SIOG). Both SIOG, WHO and DGS (Portuguese National Health Administration) give physicians guidelines to prepare them to answer adequately to elderly cancer patients. As developed societies, we cannot neglect elders or risk failing as a civilisation (19).

COVID-19 pandemic is a challenge to medical and social response, which drive health institutions' administrations to coordinate different arrangements. These new public health policies should lead the way to an adequate resolution to specific questions concerning the pandemic. It must still have the ability to implement those policies and procedure plans promptly. Innovation in healthcare amongst a pandemic scenario will surely produce fundamental changes for the future (20).

Medical professionals consider themselves specialists, not protagonists. During this battle, physicians and nurses have succumbed as soldiers of the latest biological warfare. Given this “combatant” status, we acknowledge that in a modern society, the public healthcare specialists' assignment should be reassessed (18).

## Author Contributions

VF conceived the subject and managed the entirety of the study including data entry and writing. JC supervised the study and provided relevant subject matter consultation and advice. RJ provided valuable assistance with literature review and study design.

## Conflict of Interest

The authors declare that the research was conducted in the absence of any commercial or financial relationships that could be construed as a potential conflict of interest.
